# A hybrid DenseNet121-random vector functional link (RVFL) approach for plant leaf classification

**DOI:** 10.1038/s41598-026-58360-x

**Published:** 2026-06-30

**Authors:** Upendra Mishra, Himanshi Chaudhary

**Affiliations:** 1 Department of Computer Science and Engineering, National Institute of Technology Arunachal Pradesh, Jote, Papum Pare, Arunachal Pradesh, 791113 India; 2 Department of Computer Science and Engineering, Krishna Institute of Engineering & Technology (KIET), Ghaziabad, Delhi-NCR, Uttar Pradesh India

**Keywords:** RVFL, DenseNet121, Binary classification, Engineering, Mathematics and computing

## Abstract

The Random Vector Functional Link (RVFL) network provides an efficient and quick method of training feedforward single hidden neural networks. It solves major disadvantages of classical neural networks, i.e. dealing with slow convergence and overfitting by exploiting fixed random weights and closed-form output computation. DenseNet121 architecture has huge potential in the artificial intelligence field and more specifically in object recognition tasks. The major drawback, however, is its inability to provide insights into its intermediate levels of analysis of classification, which is why this algorithm offers limited flexibility to analyze the training procedure. To resolve this problem, we offer a combined solution by integrating DenseNet121 with RVFL. Within the framework described, deep features are obtained in relation to the input leaf pictures with the pre-trained DenseNet121 model that captures important texture and contour structure. To, firstly, minimize the dimension of these features and, secondly, remove superfluous redundancies, Principal Component Analysis (PCA) is used, and only the most informative components are retained. This smaller feature set is then marked to the RVFL classification, whose responsibility is a final classification. Understanding how the proposed DenseNet121-RVFL approach performs, we have conducted some comparative experiments against several baseline classifiers, such as DenseNet121- Support Vector Machine, DesnseNet121-Twin Support Vector Machine, DenseNet121-Extreme Learning Machine, DenseNet121 and DenseNet 121-Kernel Ridge Regression. The findings of the experiment reveal that the hybrid DenseNet121-RVFL model has the best effect in comparison to the other methods, as it provided the highest accuracy of 94.45, F1-Score is 0.955, Geometeric Mean(G-Mean) of 0.898 and Area Under Curve (AUC) of 0.961 on the test dataset.

## Introduction

In the last few years, the identification and classification of plant species in the agriculture sector are significant sources of concern since there are approximately 50 thousand species that are yet to be discovered. There are enormous varieties of plant species supported by the Earth, and a substantial percentage of the plants have great therapeutic uses. There is however a classification of some as threatened and others as having different levels of toxicity to human beings. Moreover, the entire range of values, which are related to many plant species, is not well-known. Plants are essential in the food chain and serving a lifeline for human communities and the foundation of life in the country. Being an agrarian population, the populace in India is largely dependent on profitable crops. In addition to nourishment, plants are sources of numerous materials to humanity, and different parts like flowers, fruits, leaves, and roots have seen great applications across different cultures worldwide. The risk of plant infection is huge as it not only destroys a particular plant but leads to great issues in agriculture and affects the economic prosperity of a state. With the advancement of computer vision, automated methods for plant identification have gained substantial attention.

Convolutional Neural Networks (CNNs) such as AlexNet, VGGNet and ResNet seem to be performing better than ever when it involves image classification, such as identifying plant leaf diseases. Recently, neural network-based methods based on deep learning(DL) and machine learning(ML) have made significant progress in detecting plant diseases. Traditional methods relied on handcrafted features and classical classifiers such as SVM and k-Nearest Neighbours (k-NN), etc. Although these approaches showed moderate success, their performance heavily depended on domain-specific feature engineering, which is often time-consuming and lacks generalizability across datasets. Mohanty et al.^1^ used DL models like AlexNet and Google Net on the Plant Village dataset and achieved high accuracy in identifying plant diseases. Similarly, Ferentinos^2^ evaluated various CNN architectures, including LeNet, AlexNet and VGGNet, demonstrating their effectiveness in real-time agricultural environments. The study by Srivastava et al.^3^ has made a new approach to leaf classification based on the SVM with an emphasis on shape-based representation of features. Mishra et al.^4^ proposed an enhancement of a transfer learning method of using ResNet and IFRVFL to classify the leaf nodes. Mokhtar et al.^5^ have established an approach, which applies SVM to recognise and identify a diseased tomato leaf. The authors provided an elaborate classification methodology using Multi-class TWSVM, which extends the classical Twin SVM. Regression^6^and classification^7–9^, problems have been dealt with well in previous works.

Arora and Gupta.^10^ proposed an Intuitionistic Fuzzy Twin Proximal SVM (IFTP-SVM) to enhance classification performance under uncertainty. The model integrates intuitionistic fuzzy theory with twin proximal SVM to construct more robust and flexible decision boundaries. Their approach demonstrates improved accuracy and reliability, particularly in complex EEG signal classification tasks.Extending this idea, they further developed a multi-category weighted least squares twin SVM with fuzzy hyperplane for brain tumor classification^11^. Both approaches leverage fuzzy theory to enhance robustness and generalization in complex biomedical datasets. These studies highlight the effectiveness of integrating fuzzy logic with twin SVM frameworks for improved classification performance.More recently, Arora et al.^12^ proposed a relative density and non-membership-based intuitionistic fuzzy twin SVM to specifically address class imbalance issues, demonstrating enhanced robustness and generalization across complex datasets.

Sardogan et al.^13^ constructed a CNN-based model to diagnose tomato leaf diseases by training and validating a dataset consisting of different tomato leaf pictures (500 in total) with four different symptoms. In^14^, it was shown that a deep CNN model of improved performance can be proposed by including Inception-V2 and also Batch Normalisation (BN). The authors of^15^ proposed a dual-path deep CNN which combines shape-texture features with shape-context marginalized^16^. Also, Chaki et al.^17^ discussed a schema plant species classification based on leaf and examined the shape, texture, and colour features. To address data and computational limitations, transfer learning has been widely adopted. Pre-trained networks such as ResNet, Inception and DenseNet have been used as feature extractors, fine-tuned for specific agricultural tasks. While CNNs are powerful in feature extraction, the use of shallow classifiers on top of deep features has shown promising results. Deep feature extractors used in conjunction with an ML-based classifier, including SVM, Random Forests, and Extreme Learning Machines (ELMs), have proved highly accurate but much faster at training^18,19^. The fast-training single-layer feed-forward model, RVFL neural network, has also been found as be lightweight with a good generalizing property^20^. Mishra et al.^21^ introduced ifrvflc by combining RVFL and Intuitionistic fuzzy to overcome the issue of RVFL with noisy data. Mishra et al.^22^ proposed ResNet50-intuitionistic fuzzy RVFL for leaf classification. It is designed to be resource-efficient due to its capability to process necessary deep features in a non-iterative way onto the backpropagation process. Mishra et al.^4^ proposed an angle-based twin RVFL classifier that significantly improves classification accuracy with reduced computational complexity^4^. Altunay and Albayrak developed a hybrid CNN–LSTM-based intrusion detection system that effectively detects cyber-attacks in industrial IoT networks^23^. Salini et al.^24^ introduced a deep hybrid classification model that achieves high accuracy in leaf disease detection for underground crop plants^24^. Gawali and Gunjal presented a CNN–RNN-based deep learning algorithm for efficient context-based image analysis^25^.

This paper will summarise the main contributions as follows:

i. DenseNet121-RVFL model is proposed that integrates DenseNet121 for deep feature extraction using RVFL for efficient leaf image classification.
ii. The proposed DenseNet121–RVFL model achieves improved computational efficiency compared to conventional deep learning classifiers.
iii. The DenseNet121-RVFL has strong generalizability in comparison to DenseNet121-SVM, DenseNet121-TSVM, DenseNet121-ELM, DenseNet121 and DenseNet121-KRR for leaf classification.
iv. The proposed approach provides a lightweight and scalable solution suitable for real-time agricultural applications.

The organisation of the rest of the part is as follows: In Section Related models, the related work is discussed. Section Proposed methodology introduces the proposed methodology in detail. Section Experimental setup discusses the experimental setup. In Section Results and discussion, experimental outcome are discussed. In Section Friedman test and Nemenyi post-hoc test, Statistical Tests are discussed. Finally, Section Conclusion offers concluding remarks and future research.

## Related models

Consider a training dataset $\textit{D}=\{(x_i,y_i)\}_{i=1}^{r}$, where each input sample $x_i\in \mathbb {R}^{s}$ and the corresponding class label $y_i\in Y=\{ -1,+1 \}$ .The dataset consists of $r$ training samples and $s$ features. Let the positive samples be represented by $M_1 \in \mathbb {R}^{r_1 \times s}$, and the negative samples by $M_2 \in \mathbb {R}^{r_2 \times s}$.The full data matrix of training samples is denoted as $M=[x_1,x_2,\ldots ,x_r]^T$. The kernel function $k(\cdot ,\cdot )$ is defined as $K(x^T,M^T)=\bigl (k(x,x_1),k(x,x_2),\ldots ,k(x,x_r)\bigr )$ where for any $x \in \mathbb {R}^s$, the kernel is computed as $k(x, x_j) = \varphi (x)^T \varphi (x_j)$, with $\varphi (\cdot )$ denoting the feature mapping.

### SVM

SVM is a well-established supervised learning technique grounded in statistical learning theory^26,27^. An optimal hyperplane is constructed by maximizing the separation margin between two classes in the transformed feature space.Given a training sample set $\{(x_i, y_i)\}_{i=1}^N$, where $x_i \in \mathbb {R}^d$ and class labels $y_i \in \{-1, +1\}$. The SVM model creates a convex optimization model that is used to maximize the minimum gap and to reduce the classification errors using slack variables.

The optimal formulation of SVM is given by:

1 $$\begin{aligned} \min _{w, b, \xi } \ \frac{1}{2}\Vert w\Vert ^2 + C \sum _{i=1}^N \xi _i \end{aligned}$$

subject to:

2 $$\begin{aligned} y_i (w^\top x_i + b) \ge 1 - \xi _i, \quad \xi _i \ge 0. \end{aligned}$$

Here, *w* and *b* define the separating hyperplane, $\xi _i$ represents the hinge loss, and *C* controls the trade-off between model complexity and empirical risk.The dual form can be obtained by adding Lagrange multipliers and applying the Karush KuhnTucker (KKT) conditions.This yields a sparse solution in which the classification boundary is influenced by only a limited number of support vectors. Although SVM is computationally efficient in terms of its generalization capability, it is ineffective with large data volumes and requires sensitive choice of kernel and regularization parameter settings.

### TSVM

To mitigate some of the limitations of conventional SVM, particularly its computational burden, the TSVM was introduced as an efficient alternative. In contrast to SVM, which constructs a single optimal hyperplane, TSVM determines two non-parallel hyperplanes, where each is nearer to one class and distant from the opposing class^28^.

Let $M_1$ and $M_2$ are the data matrices related to the two classes. TSVM seeks two hyperplanes defined as:

3 $$\begin{aligned} K(M_1, M^T)\mu _1 + b_1 \textbf{e}_1 = 0, \end{aligned}$$

4 $$\begin{aligned} K(M_2, M^T)\mu _2 + b_2 \textbf{e}_2 = 0, \end{aligned}$$

where $K(\cdot ,\cdot )$ represents the kernel function and $\textbf{e}_1$, $\textbf{e}_2$ are vectors of ones.

The optimization problem for the first hyperplane is formulated as:

5 $$\begin{aligned} \min _{\mu _1, b_1, \tau _1} \frac{1}{2} \left\| K(M_1, M^T)\mu _1 + b_1 \textbf{e}_1 \right\| ^2 + C_1 \textbf{e}_2^T \tau _1, \end{aligned}$$

subject to:

6 $$\begin{aligned} - \left( K(M_2, M^T)\mu _1 + b_1 \textbf{e}_2 \right) + \tau _1 \ge \textbf{e}_2, \quad \tau _1 \ge 0. \end{aligned}$$

A similar optimization problem is solved for the second hyperplane. TSVM is computationally efficient as it will divide the problem into two smaller quadratic programming problems(QPP) as opposed to a large optimization problem.^29^. For prediction, a test sample is assigned to the class corresponding to the closer hyperplane based on normalized distance:

7 $$\begin{aligned} \text {class}(x) = \text {sign}\left( \frac{|K(x^T, M^T)\mu _1 + b_1|}{\Vert \mu _1\Vert } - \frac{|K(x^T, M^T)\mu _2 + b_2|}{\Vert \mu _2\Vert } \right) . \end{aligned}$$

### ELM

ELM is a fast learning algorithm for single-layer feedforward neural networks (SLFNs). The primary distinction of ELM lies in its training strategy, where the input weights and hidden layer biases are randomly generated and kept fixed, while the output weights are analytically computed^30^.

Let *H* denote the hidden layer output matrix generated using nonlinear activation functions. The ELM output is defined as:

8 $$\begin{aligned} y = H \mu , \end{aligned}$$

where $\mu \in \mathbb {R}^{L+s}$ represents the output weight vector. For classification tasks, the discriminant rule is expressed as:

9 $$\begin{aligned} f(x) = \text {sign}(h(x)\mu ). \end{aligned}$$

ELM offers extremely fast training and avoids iterative weight updates, making it suitable for large datasets. However, its performance is sensitive to the random initialization of hidden nodes and the choice of activation functions.

### KRR

KRR is a kernel-based learning method that combines ridge regression with kernel functions to address nonlinear classification problems^31^. Unlike SVM, KRR does not employ margin maximization or inequality constraints, resulting in a simpler optimization framework.

The dual optimization problem of KRR is formulated as:

10 $$\begin{aligned} \min _{\mu } \; C \Vert \mu \Vert ^2 + \tau ^2, \end{aligned}$$

subject to:

11 $$\begin{aligned} y - \phi (M_0)^T \mu = \tau , \end{aligned}$$

where $\phi (\cdot )$ denotes the feature mapping induced by the kernel, $\tau$ is the residual error, and *C* is the regularization parameter.

For a new input sample *x*, classification is performed as:

12 $$\begin{aligned} \text {class}(x) = \operatorname {sign}(\phi (x)^T \mu ). \end{aligned}$$

KRR provides closed-form solutions and stable convergence but may suffer from overfitting when regularization is not carefully controlled.

### RVFL

RVFL networks belong to the family of single-layer feedforward neural networks and are recognized for their simplicity and computational efficiency. Unlike traditional neural networks, RVFL incorporates direct connections between the input and output layers in addition to the hidden layer^32^.

Given an input vector $x \in \mathbb {R}^d$, the hidden layer output is computed using a nonlinear activation function:

13 $$\begin{aligned} h = g(W_r x + b_r), \end{aligned}$$

where $W_r \in \mathbb {R}^{N \times d}$ and $b_r \in \mathbb {R}^N$ are randomly initialized and fixed parameters.

The final network output is given by:

14 $$\begin{aligned} y = W_0 h + b_o, \end{aligned}$$

where $W_0$ and $b_o$ denote the trainable output weights and bias. These parameters are typically learned using least squares estimation, resulting in rapid training and reduced risk of local minima. RVFL has demonstrated strong generalization performance in classification tasks due to its randomization and linear output learning mechanism.

### Comparative discussion

The reviewed learning models differ significantly in terms of formulation, computational complexity, and learning paradigms. SVM establishes a single maximum-margin hyperplane and offers strong theoretical generalization guarantees; however, its training complexity increases considerably with large-scale datasets. TSVM addresses this limitation by constructing two non-parallel hyperplanes through smaller QPP, thereby reducing computational burden while maintaining competitive classification performance. KRR provides a simpler optimization framework with closed-form solutions but lacks the margin-based robustness characteristic of SVM-based methods.

Neural-network-based approaches such as ELM and RVFL adopt randomized learning strategies that eliminate iterative weight updates, resulting in substantially faster training. ELM relies solely on randomly generated hidden representations, which can lead to performance variability, whereas RVFL enhances stability by incorporating direct input-to-output connections alongside hidden-layer transformations. Overall, margin-based classifiers demonstrate strong theoretical robustness, while randomization-based neural models offer superior efficiency and scalability. Table 1 shows the comparative evaluation of related models.

**Table 1:** Comparative evaluation of related models.

Model	Learning principle	Optimization strategy	Computational characteristics	Key strengths and limitations
SVM	Margin maximization using a single separating hyperplane	Convex quadratic programming with inequality constraints	Computationally expensive for large datasets; kernel choice impacts scalability	Strong theoretical generalization; sensitive to parameter tuning and kernel selection
TSVM	Two non-parallel hyperplanes, each proximal to one class	Two smaller QPP	Lower computational cost than SVM; suitable for medium-scale data	Improved efficiency; classification depends on distance normalization accuracy
KRR	Regularized least squares in kernel-induced feature space	Closed-form solution via ridge regression	Efficient training; memory-intensive for large kernel matrices	Simple optimization; lacks margin-based robustness
ELM	Randomized hidden layer with analytical output weight computation	Linear least squares solution	Extremely fast training; performance varies with random initialization	High training speed; limited control over hidden representations
RVFL	Random hidden mapping with direct input-to-output connections	Linear regression-based output learning	Efficient and stable; scalable to large datasets	Enhanced generalization via direct links; randomness may affect reproducibility

## Proposed methodology

**Fig. 1:**
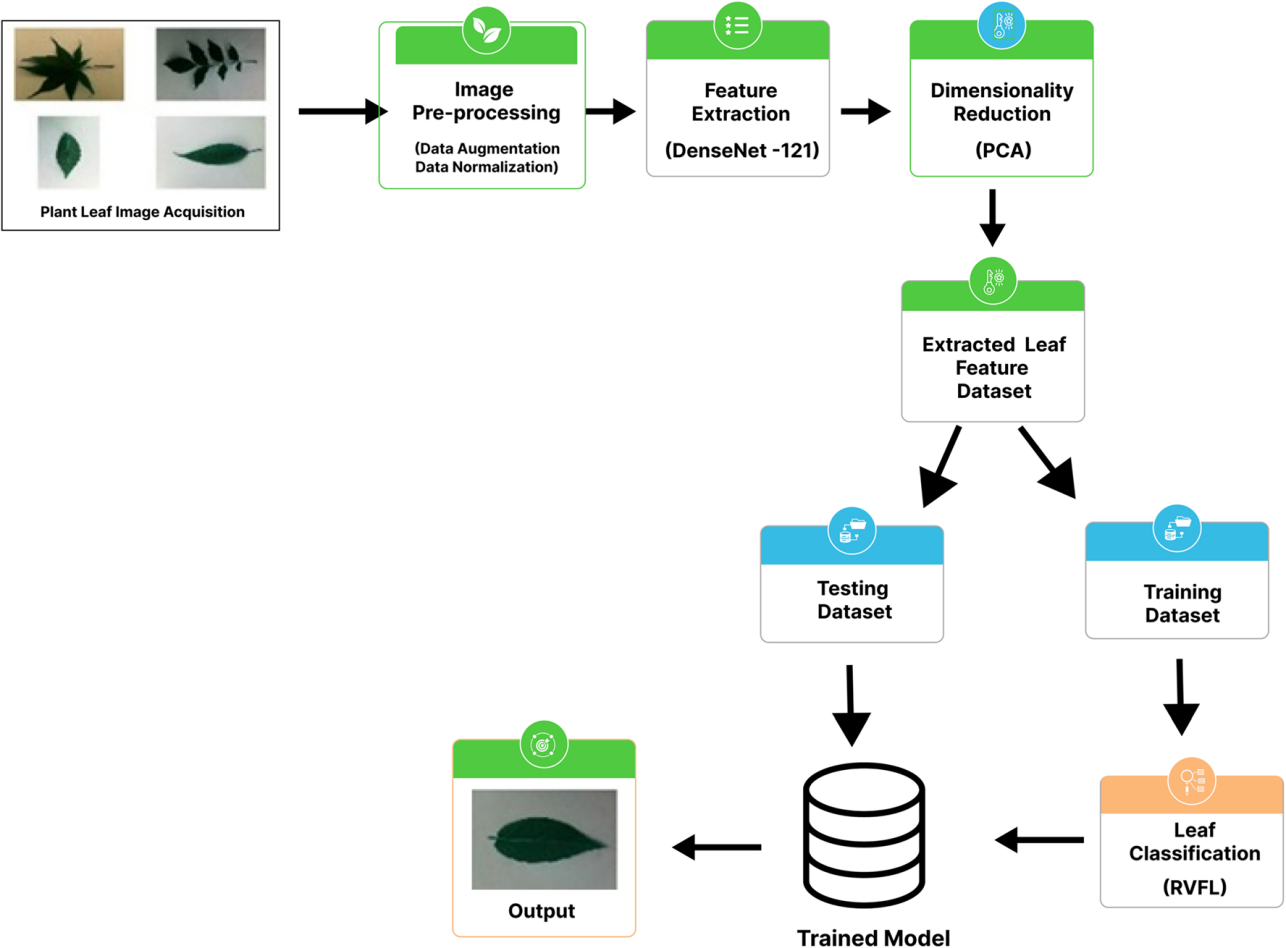
Proposed DenseNet121-RVFL architecture visualization.

**Fig. 2:**
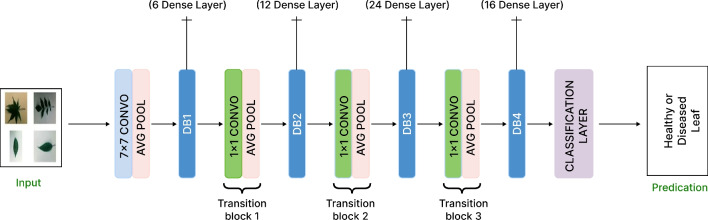
DenseNet121 architecture visualization.

In this study, a generalized model based on hybrid DL and ML and the classification of plant leaves into healthy or diseased is presented. This system exploits feature extraction in terms of images and advanced classification in terms of high accuracy of plant health estimation, Originally 1024 features are extracted from Densenet121. After extracting a 1024-dimensional feature vector from the global average pooling layer of DenseNet121, PCA was applied to obtain a lower-dimensional representation of the data.The principal components accounting for 30% of the total variance were selected for further analysis.Then these 30 components were used as input to the RVFL classifier. In Figure.1 proposed methodology of DenseNet121-RVFL is shown below:

The Proposed Algorithm contains a number of Steps that are illustrated below:

1. Read plant leaves data of healthy and disease.
2. The data preprocessing stage involved several operations, including data cleaning, noise removal, data filtering, normalization, feature scaling, transformation, and handling of missing values.
3. Pretrained DenseNET-121 is used to extract features and it is depicted in Figure. 2.
4. Conduct Dimensionality reduction by means of PCA to get more significant features.
5. The extracted feature dataset was separated into training and testing samples.
6. Training the model on the training dataset.
7. Check the model with a test dataset and identify the accuracy of the model.

### Plant leaf image acquisition

The initial stage of the framework is a collection of a diversified data set comprising plant leaf images. The pictures are also taken in different kinds of lighting and environmental conditions with a view to guaranteeing the robustness of the models. Under the dataset, there are healthy samples as well as diseased samples of various plant species.

### Preprocessing

This is done by a preprocessing step to elevate the accuracy of the input data and simplify the more complicated aspects of the calculations. This contains image resizing, noise removal, normalization and enhancement of contrast. Preprocessing makes the images similarly organised and geared towards the following phase of processing.

### Feature extraction using DenseNet121

After preprocessing, the deep feature gets extracted based on the DenseNet121 architecture of the densely connected convolutional neural network, which is characterised by the economy of time and precision in its efficiency as an image recognition task. In this case, DenseNet121 is treated as a feature extractor (but not a classifier) by discarding the fully-connected layers that sit at the top of a network, and keeping the high-level spatial features produced in the convolutional layers.In Figure.2 Architecture of DenseNet121 is shown.

### Dimensionality reduction using PCA

The dimensionality of the extracted features is large; hence, to reduce the feature space, PCA) is used.PCA helps in removing redundant and less significant features, leading to improved training efficiency and reduced risk of overfitting.PCA functions well when the features of the inputs are very correlated. It involves four main steps:

Step 1: Firstly, the initial variables are scaled in such a way that they contribute equally to the analysis.

Step 2: Covariance Matrix Calculation: The aim of this step is to find out how the variables belonging to the input dataset deviate out of their mean, in relation to each other. In a bid to evaluate the dispersion of data in the leaf image dataset, the first step taken is to compute the variance, which is done using the following formula:

15 $$\begin{aligned} \textrm{Var}(p) = \sigma ^2 = \frac{1}{n} \sum _{i=1}^{n} (z_{ij} - \mu _j)^2 \end{aligned}$$

where *n* is the number of observations, $z_{ij}$ denotes the value of the $j^{\text {th}}$ feature for the $i^{\text {th}}$ sample, $\mu _j$ represents the mean of the $j^{\text {th}}$ feature, and $\sigma ^2$ indicates the variance. The covariance matrix is computed using the following formula:

16 $$\begin{aligned} \textrm{Cov}(p, q) = \frac{1}{n - 1} \sum _{i=1}^{n} (p_{ij} - \mu _j)(q_{ij} - \mu _j) \end{aligned}$$

Step 3: Calculation of the eigenvalues and eigen vectors of the covariance that identify the principal components.

Step 4: Selection of a feature vectors: This step consists of determining what features we keep and which ones we drop (based on their eigenvalues) usually the ones with smaller eigenvalues are dropped. The other elements are then grouped together in a matrix which is referred to as feature vector. First, the eigenvalues are to be rotated by an orthogonal rotation by the equation presented below:

17 $$\begin{aligned} \det (A - \lambda I) = 0 \end{aligned}$$

where *A* denotes the covariance matrix, $\lambda$ is the eigenvalues, *I* denotes the identity matrix, and $\det (\cdot )$ is the determinant.

### Classification using RVFL network

PCA is applied to the data in order to derive the Reduced Feature Vectors, and the latter are input to an RVFL network and classified.RVFL refers to a single-layer forward type of neural networks that is fast and known to have generalization ability. It is best applied in cases of limited labelled data as well as in cases where non-linear patterns are complex in nature.

**Fig. 3:**
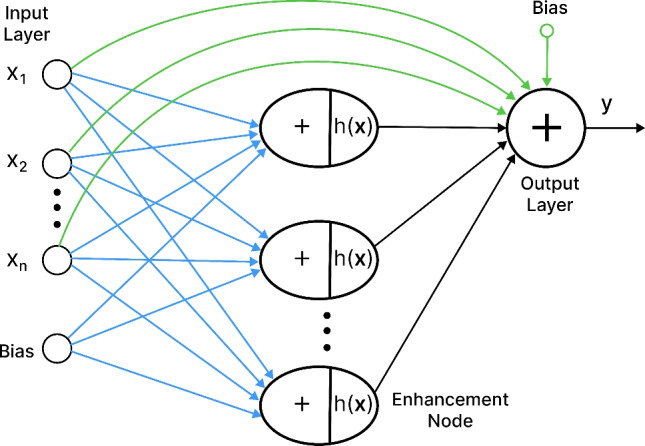
RVFL architecture.

In Figure.3 the detailed architecture of RVFL is discussed.Here, the red lines depict the direct feature mappings between the input and the output layer. The weights of the blue connections are randomly initialised in some predefined range and fixed throughout the training. As a result, the output weights of the red and black do not determine the possible estimations^33–35^.

## Experimental setup

The experimental analysis was performed in MATLAB R2023b on a Windows 11 machine configured with an Intel Core i9 processor 1.20 GHz and 32 GB RAM. Moreover, to validate the effectiveness of the DenseNet121-RVFL model, an additional leaf dataset containing samples from different tree varieties was incorporated into the study. Furthermore, a 10-fold cross-validation strategy was employed to identify the optimal parameter settings.The classifiers use a Gaussian Radial Basis Function (RBF) nonlinearity, which is written as follows:

18 $$\begin{aligned} K(x_a, x_b) = \exp \left( -\mu \, \Vert x_a - x_b \Vert ^2 \right) . \end{aligned}$$

where $x_a$ and $x_b$ are random input data. The range of all parameters involved in the experiments is presented in Table 2.

**Table 2:** Attribute ranges for each presented classifiers.

Parameters	SVM	TSVM	ELM	KRR	RVFL
*C*	$\{10^{-6}, \ldots , 10^{0}\}$	$\{10^{-6}, \ldots , 10^{0}\}$	$\{10^{-6}, \ldots , 10^{0}\}$	$\{10^{-5}, \ldots , 10^{5}\}$	$\{10^{-6}, \ldots , 10^{0}\}$
$\mu$	$\{2^{-5}, \ldots , 2^{5}\}$	-	-	$\{2^{-5}, \ldots , 2^{5}\}$	-
*L*	-	$\{20, 50, 100, 500, 1000\}$	$\{20, 50, 100, 500, 1000\}$	-	$\{20, 50, 100, 500, 1000\}$
$c_1$	–	-	-	-	-
$c_2$	-	-	-	-	-

## Results and discussion

The efficiency and generalization capability of the DenseNet121-RVFL model are analyzed by using Plant leaf Datasets.The dataset of the leaves comprises 854 images which include various types of leaves like mango and the guava. These images are extracted from Kaggle Leaf Image Dataset.For experimental evaluation, 70% of the dataset was utilized for training, while the remaining 30% was reserved for testing and validation.

Table 3 shows the accuracy of models where one can note that the proposed model has the lowest mean rank. Similarly, Table 4 shows F1-Score, Table 5 shows G-Mean, and Table 6 shows AUC of the models.Additionally, the results indicate that the proposed DenseNet121-RVFL model outperforms other existing models, including DenseNet121-SVM, DenseNet121-TSVM, DenseNet121-ELM, DenseNet121, and DenseNet121-KRR.

**Fig. 4:**
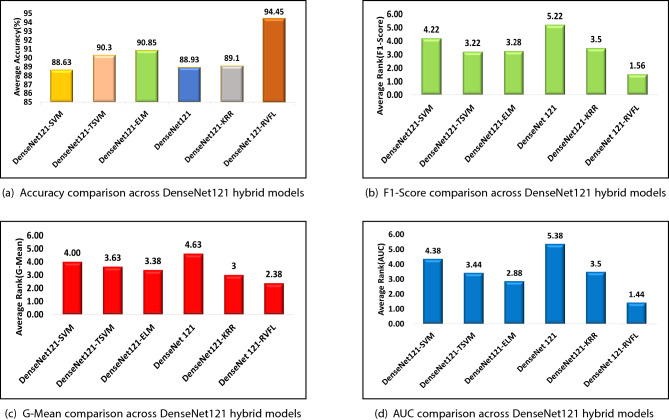
Performance comparison of DenseNet121-RVFL with other hybrid models using different evaluation metrics.

**Accuracy:** As shown in Table 3, the proposed DenseNet121-RVFL achieves the highest average accuracy (94.45%), surpassing DenseNet121-SVM (88.63%), DenseNet121-TSVM (90.3%), DenseNet121-ELM (90.85%), DenseNet121 (88.93%), and DenseNet121-KRR (89.1%). Figure 4a demonstrates the visualization of the accuracy comparison of DenseNet121-RVFL with some existing models.

19 $$\begin{aligned} \text {Accuracy} = \frac{TP + TN}{TP + TN + FP + FN} \end{aligned}$$

Here, TP, TN, FP, and FN represent the number of True Positives, True Negatives, False Positives, and False Negatives, respectively.

**F1-Score:** The F1-score shown in Table 4 measures the trade-off between precision and recall, making it particularly appropriate for imbalanced classification problems. The proposed DenseNet121-RVFL model attained an average F1-score of 0.955, outperforming DenseNet121-ELM and DenseNet121-TSVM, which achieved average F1-scores of 0.941 and 0.943, respectively. Furthermore, Figure 4b illustrates the comparative visualization of F1-scores between DenseNet121-RVFL and several existing models.

20 $$\begin{aligned} \text {F1-Score} = \frac{2 \times \text {Precision} \times \text {Recall}}{\text {Precision} + \text {Recall}} \end{aligned}$$

**G-Mean:** The G-Mean metric presented in Table 5 assesses the balance between sensitivity and specificity. The proposed model consistently achieved the highest average G-Mean value of 0.898 along with the best mean rank of 2.38. Furthermore, Figure 4c illustrates the comparative visualization of the G-Mean performance of DenseNet121-RVFL against several existing models.

21 $$\begin{aligned} \text {G-Mean} = \sqrt{\text {Recall} \times \text {Specificity}} \end{aligned}$$

where

22 $$\begin{aligned} \text {Precision} = \frac{TP}{TP + FP} \qquad \text {and} \qquad \text {Specificity} = \frac{TN}{TN + FP} \end{aligned}$$

**AUC:** Table 6 presents the AUC metric, which measures how well the model differentiates between positive and negative classes across various thresholds. The proposed DenseNet121-RVFL model demonstrates superior performance, attaining the highest average AUC of 0.961 along with the best mean rank of 1.44. Figure 4d demonstrates the visualization of the AUC comparison of DenseNet121-RVFL with some existing models.

23 $$\begin{aligned} \text {AUC} = \int _{0}^{1} \text {TPR}(x)\, d(\text {FPR}(x)) \end{aligned}$$

where

24 $$\begin{aligned} \text {TPR} = \frac{TP}{TP + FN} \qquad \text {and} \qquad \text {FPR} = \frac{FP}{FP + TN} \end{aligned}$$

**Fig. 5:**
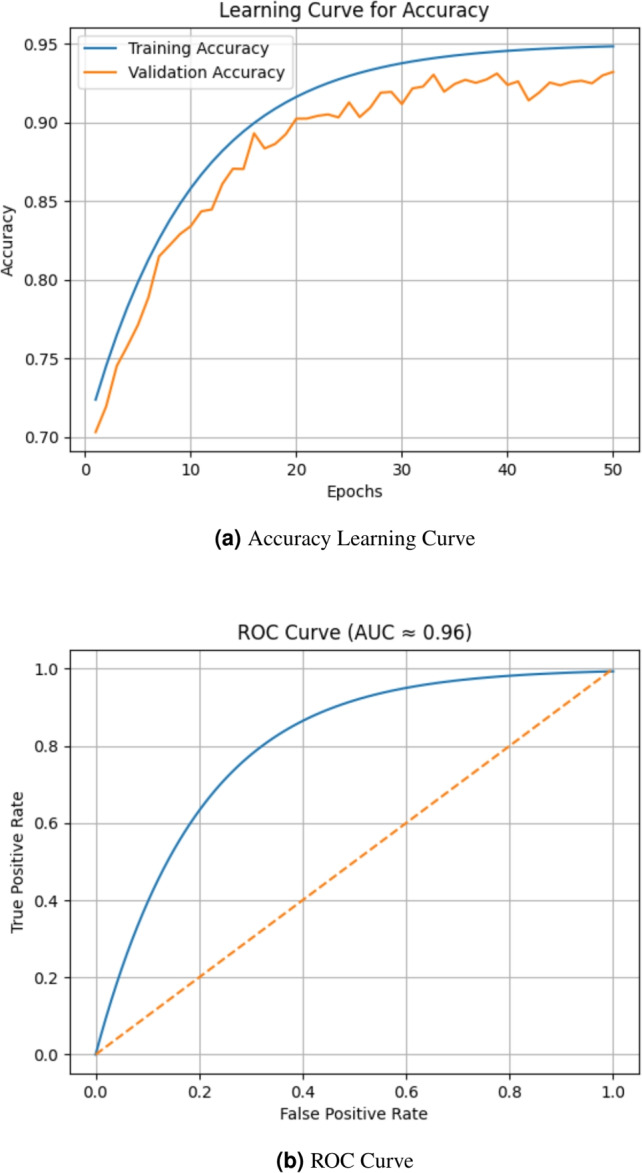
Performance evaluation plots.

Learning Curve Analysis:

i) Accuracy Learning Curve: The learning curve depicts the progression of training and validation accuracy across different epochs. It is evident that both curves gradually increase and eventually converge, indicating that the proposed DenseNet121-RVFL model exhibits stable learning behavior with minimal overfitting. The same can be seen in Figure 5.

ii) ROC Curve: The ROC curve highlights the predictive performance of the proposed model. The curve remains close to the upper-left corner, reflecting a high true positive rate along with a low false positive rate. In addition, the obtained AUC value of approximately 0.96 validates the excellent discriminative ability of the model. The same can be seen in Figure 5.

**Table 3:** Comparative accuracy analysis of DenseNet121-RVFL with baseline models.

Datasets (train $\times$ test)	DenseNet121-SVM	DenseNet121-TSVM	DenseNet121-ELM	DenseNet121	DenseNet121-KRR	DenseNet121-RVFL
	**Accuracy (%)**					
	**Rank**					
	**Time (s)**					
LEMON (165 $\times$ 71)	81.71	86.85	85.43	84.85	87.14	**94.43**
	6	3	4	5	2	**1**
	0.01332	0.01722	0.000833025	0.00918	0.00041	0.0004907
GUAVA (294 $\times$ 126)	93.93	91.36	94.24	93.36	91.40	**96.56**
	3	6	2	4	5	**1**
	0.02942	0.0389	0.00213294	0.03909	0.01194	0.0025277
JATROPHA (180 $\times$ 78)	91.79	**94.74**	88.26	87.74	84.42	90.78
	2	**1**	4	5	6	3
	0.01322	0.01782	0.00304308	0.01519	0.001683	0.003521
MANGO (304 $\times$ 131)	91.70	91.91	93.62	90.91	93.23	**98.31**
	5	4	2	6	3	**1**
	0.4088	0.546	0.00522347	0.49084	0.06005	0.0670615
CHINAR (157 $\times$ 67)	89.55	89.39	90.89	90.39	82.96	**91.61**
	4	5	2	3	6	**1**
	0.0058	0.01192	0.000966459	0.01238	0.001148	0.0006345
ARJUN (317 $\times$ 136)	88.55	89.19	89.19	84.19	**94.96**	93.67
	5	3.5	3.5	6	**1**	2
	0.005971	0.01114	0.00111206	0.01182	0.000698	0.00035942
JAMUN (437 $\times$ 188)	88.17	89.92	93.59	92.92	89.84	**96.62**
	6	4	2	3	5	**1**
	0.01788	0.0274	0.00153175	0.02924	0.00271	0.00391574
ORANGE (398 $\times$ 171)	88.95	89.06	91.59	87.06	88.84	**93.62**
	4	3	2	6	5	**1**
	0.007124	0.01114	0.000944002	0.01199	0.001071	0.00059356
**Mean Accuracy**	**88.63**	**90.30**	**90.85**	**88.93**	**89.10**	**94.45**
**Mean Rank**	**4.38**	**3.69**	**2.69**	**4.75**	**4.13**	**1.38**

**Table 4:** F1 Score comparison of DenseNet121-RVFL with baseline models.

Datasets (train $\times$ test)	DenseNet121-SVM	DenseNet121-TSVM	DenseNet121-ELM	DenseNet121	DenseNet121-KRR	DenseNet121-RVFL
	**F1-Score**					
	**Rank**					
LEMON (165 $\times$ 71)	0.938	**0.987**	0.901	0.932	0.926	0.961
	3	1	6	4	5	2
GUAVA (294 $\times$ 126)	0.953	0.962	0.945	0.898	0.931	**0.972**
	3	2	4	6	5	1
JATROPHA (180 $\times$ 78)	0.948	0.940	**0.969**	0.909	0.915	0.932
	2	3	1	6	5	4
MANGO (304 $\times$ 131)	0.946	0.953	0.965	0.968	0.958	**0.993**ara>
	6	5	3	2	4	1
CHINAR (157 $\times$ 67)	0.938	0.936	0.944	0.863	0.941	**0.960**
	4	5	2	2	3	1
ARJUN (317 $\times$ 136)	0.918	0.926	0.926	0.923	**0.951**	0.937
	6	3.5	3.5	5	1	2
JAMUN (437 $\times$ 188)	0.912	0.901	0.938	0.887	0.941	**0.952**
	4	5	3	6	2	1
ORANGE (398 $\times$ 171)	0.932	0.941	0.938	0.912	0.941	**0.952**
	5	2.5	4	6	2.5	1
**Mean F1 Score**	**0.936**	**0.943**	**0.941**	**0.912**	**0.938**	**0.955**
**Mean Rank**	**4.22**	**3.22**	**3.28**	**5.22**	**3.50**	**1.56**

**Table 5:** G-Mean comparison of DenseNet121-RVFL with baseline models.

Datasets (Train $\times$ Test)	DenseNet121-SVM	DenseNet121-TSVM	DenseNet121-ELM	DenseNet121	DenseNet121-KRR	DenseNet121-RVFL
	**G-Mean**					
	**Rank**					
LEMON (165 $\times$ 71)	0.867	0.807	0.861	0.827	0.891	**0.910**
	3	6	4	5	2	1
GUAVA (294 $\times$ 126)	**0.953**	0.862	0.845	0.812	0.932	0.892
	1	4	5	4	2	3
JATROPHA (180 $\times$ 78)ara>	0.848	0.887	0.839	0.798	0.921	**0.951**
	4	3	5	6	2	1
MANGO (304 $\times$ 131)	0.648	0.703	0.912	0.803	0.942	**0.952**
	6	5	3	4	2	1
CHINAR (157 $\times$ 67)	0.738	0.836	**0.924**	0.786	0.881	0.778
	6	3	1	4	2	5
ARJUN (317 $\times$ 136)	0.638	**0.936**	0.626	0.812	0.831	0.878
	5	1	6	4	3	2
JAMUN (437 $\times$ 188)	0.832	0.861	0.878	0.678	0.793	**0.900**
	4	3	2	6	5	1
POMEGRANATE (398 $\times$ 171)	0.937	0.923	**0.947**	0.941	0.901	0.921
	3	4	1	2	6	5
**Mean G-Mean**	**0.810**	**0.852**	**0.849**	**0.807**	**0.886**	**0.898**
**Mean Rank**	**4.00**	**3.63**	**3.38**	**4.63**	**3.00**	**2.38**

**Table 6:** AUC comparison of DenseNet121-RVFL with other models.

Datasets (Train $\times$ Test)	DenseNet121-SVM	DenseNet121-TSVM	DenseNet121-ELM	DenseNet121	DenseNet121-KRR	DenseNet121-RVFL
	**AUC**					
	**Rank**					
LEMON (165 $\times$ 71)	0.893	0.927	0.904	0.897	0.928	**0.934**
	6	3	4ara>	5	2	1
GUAVA (294 $\times$ 126)	0.954	0.963	0.946	0.923	**0.981**	0.972
	4	3	5	6	1	2
JATROPHA (180 $\times$ 78)	0.909	0.950	0.975	0.945	0.919	**0.985**
	6	3	2	4	5	1
MANGO (304 $\times$ 131)	0.927	0.904	**0.966**	0.921	0.939	0.964
	4	6	1	5	3	2
CHINAR (157 $\times$ 67)	0.939	0.938	0.926	0.898	0.906	**0.971**
	2	3	4	6	5	1
ARJUN (317 $\times$ 136)	0.939	0.938	0.928	0.896	0.906	**0.968**
	2	3	4	6	5	1
JAMUN (437 $\times$ 188)	0.894	0.943	**0.960**	0.923	0.942	0.943
	6	2.5	1	5	4	2.5
ORANGE (398 $\times$ 171)	0.907	0.923	0.947	0.901	0.942	**0.953**
	5	4	2	6	3	1
**Mean AUC**	**0.921**	**0.936**	**0.944**	**0.913**	**0.933**	**0.961**
**Mean Rank**	**4.38**	**3.44**	**2.88**	**5.38**	**3.50**	**1.44**

## Time complexity comparison

As shown in Table 7, traditional kernel-based methods such as SVM, TSVM, and KRR exhibit cubic time complexity, making them computationally expensive for large-scale datasets. In contrast, the proposed DenseNet121-RVFL model achieves a lower time complexity of $O(N \cdot d \cdot L)$. due to its non-iterative training mechanism, thereby ensuring faster convergence and improved computational efficiency.

**Table 7:** Time complexity comparison of different models.

Model	Training time complexity	Testing time complexity
DenseNet121-SVM	$O(N^3)$	$O(N \cdot d)$
DenseNet121-TSVM	$O(N^3)$	$O(N \cdot d)$
DenseNet121-ELM	$O(N \cdot d \cdot L + L^3)$	$O(d \cdot L)$
DenseNet121	$O(N \cdot d \cdot E)$	*O*(*d*)
DenseNet121-KRR	$O(N^3)$	$O(N \cdot d)$
**DenseNet121-RVFL (Proposed)**	$\mathbf {O(N \cdot d \cdot L + L^3)}$	$\mathbf {O(d \cdot L)}$

table 7$N$ indicates the number of training samples, $d$ indicates the feature space dimension extracted from DenseNet121, $L$ indicates the number of hidden or enhancement nodes, and $E$ refers to the training epochs.

## Parameter insensitivity

**Fig. 6:**
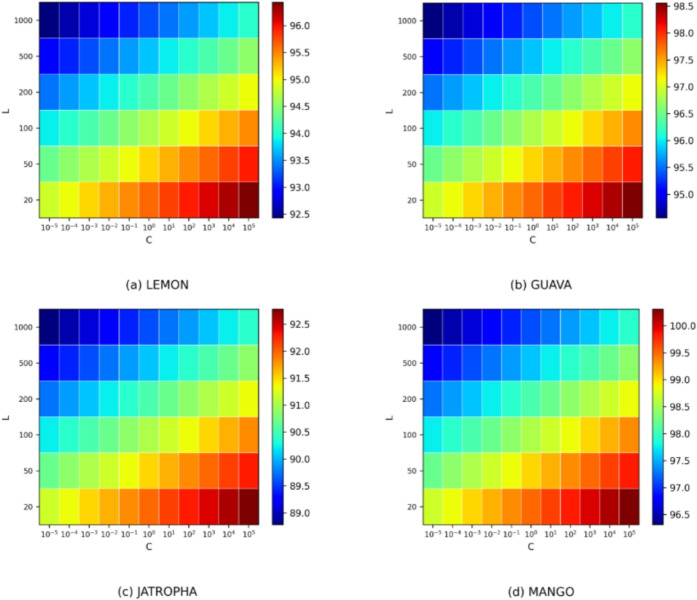
Parameter insensitivity analysis on Leaf Datasets.

Figure 6 shows the and parameter insensitivity plots of (a) Lemon (b) Guava (c) Jatropha and (d) Mango. Here L (number of hidden nodes) and C (coefficient) are two important parameters of DenseNet121-RVFL that need to be tuned before learning process begins. Grid-search technique is used here to vary L and C in range {20, 50, 100, 200, 500, 1000} and ${\{10^{5}, \ldots ,10^{-5}}\}$, respectively. From the figure, it is clear that proposed DenseNet121-RVFL is insensitive to user-defined parameters. Little change in accuracy with respect to different values of parameter can be noticed.

## Friedman test and nemenyi post-hoc test

The statistical tool used in this experiment is the Friedman test,a sound and trustworthy non-parametric test, to determine the performance of various classification models using various datasets. The test is applied to test the hypothesis of whether DenseNet121-RVFL shows statistically significant increase in terms of classification accuracy as reported in Table 2. During the Friedman ranking process , the classifier with the best performance will be ranked as 1, the second-best model will have the rank 2, and so on. The ranking is determined based on the classification accuracy obtained for each dataset. In this experiment, a total of 8 datasets and 6 classifiers are used. When the numbers of datasets *n* and classifiers *k* are sufficiently large, the Friedman statistic has a distribution that fits the $F_F$ distribution with $k - 1$ degrees of freedom. With respect to classification accuracy, the null hypothesis assumes that there is no statistically significant difference among the performance of the evaluated classifiers^36^.

Null Hypothesis $N_0$ = The performance of the assessed classifiers is not statistically different.

Alternative Hypothesis $N_1$ = There is at least one classifier whose performance difference is statistically significant with the rest.

If the Friedman test statistic $\left( \chi _F^2\right)$ exceeds the critical value, the null hypothesis is rejected.

25 $$\begin{aligned} \chi _F^2 = \frac{12n}{k(k+1)} \left[ \sum _{j=1}^{k} A_j^2 - \frac{k(k+1)^2}{4} \right] \end{aligned}$$

where $A_j$ corresponds to the average classification accuracy rank of the $j^{\text {th}}$ model.The calculation process is outlined as;

26 $$\begin{aligned} A_j = \frac{1}{n} \sum _{i=1}^{n} a_i^{\,j} \end{aligned}$$

where $a_i^{\,j}$ represents the classification accuracy rank of the $j^{\text {th}}$ classification model when evaluated on the $i^{\text {th}}$ data set. Table 3 shows the mean ranking of the classification models of the assessed models. As Iman and Davenport^37^ showed, Friedman’s $F_F$ is an overly conservative statistic and consequently proposed an alternative test statistic.

27 $$\begin{aligned} F_F = \frac{(n-1)\chi _F^2}{n(k-1) - \chi _F^2} \end{aligned}$$

This statistic follows an F-distribution with the corresponding degrees of freedom $(k - 1)$ and $(k - 1)(n - 1)$. To validate that the differences in mean ranks among the groups are statistically significant, $\chi _F^2$ and $F_F$ can be calculated as:

28 $$\begin{aligned} \chi _F^2 = \frac{12 \times 8}{6(6+1)} \left[ 4.38^2 + 3.69^2 + 2.79^2 + 4.75^2 + 4.13^2 + 1.38^2 - \frac{6(6+1)^2}{4} \right] = 19.65 \end{aligned}$$

29 $$\begin{aligned} F_F = \frac{(8 - 1)\times 19.65}{8(6 - 1) - 19.65} = 6.759 \end{aligned}$$

The statistic follows an F-distribution with $(6 - 1)$=5 and $(6 - 1)(8 - 1)$ = 35 degrees of freedom corresponding to the 6 classification models and 8 datasets, respectively. For a significance level of 0.05, the critical value for *F*(5, 35) is 2.45. The null hypothesis $N_0$ is rejected, as the critical value is lower than $F_F = 6.759$. As the null hypothesis was rejected, a post hoc analysis was carried out. In this regard, the Nemenyi test^38^ was used as the next stage of the evaluation. In the Nemenyi test, the Critical Difference (CD) is first calculated using the equations presented below:

30 $$\begin{aligned} CD = q_{\alpha } \sqrt{\frac{k(k+1)}{6n}} \end{aligned}$$

where $q_\alpha$ is the critical value

31 $$\begin{aligned} CD = 2.45 \times \sqrt{\frac{6(6+1)}{6 \times 8}} = 2.29 \end{aligned}$$

**Table 8:** Comparative evaluation of DenseNet121-RVFL against other classifiers using the Nemenyi post-hoc test.

DenseNet121-RVFL vs	DenseNet121-SVM	DenseNet121-TSVM	DenseNet121-ELM	DenseNet121	DenseNet121-KRR
Mean Rank of DenseNet121-RVFL ($P_1$)	1.38	1.38	1.38	1.38	1.38
Mean Rank of baseline models ($P_2$)	4.38	3.69	2.69	4.75	4.13
$P_2 - P_1$	3.00	2.31	1.31	3.37	2.75
CD	2.29	2.29	2.29	2.29	2.29
Comparison of $(R_2-R_1)$ and CD	$3.00>2.29$	$2.31>2.29$	$1.31<2.29$	$3.37>2.29$	$2.75>2.29$
Significance Difference	$\checkmark$	$\checkmark$	$\checkmark$	$\checkmark$	$\checkmark$

**Fig. 7:**
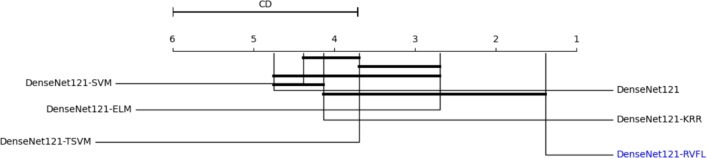
Visual analysis of classifiers applied to plant leaf data using the Nemenyi post-hoc test.

Based on the results presented in Table 8, it is evident that the proposed DenseNet121-RVFL approach demonstrates significantly superior performance compared to baseline models. From Figure 7 it is clear visible that DenseNet121-RVFL model outperforms baseline models.

## Conclusion

In this study, we proposed a hybrid classification model that combines the powerful feature extraction capabilities of the pre-trained DenseNet121 convolutional neural network with the fast and interpretable classification strength of the RVFL network. This hybridization enables the model to deliver superior accuracy and robust generalization with reduced computational overhead in binary classification tasks. Moreover, the DenseNet121-RVFL model exhibits low sensitivity to user-defined parameters across diverse datasets. By leveraging DenseNet121 for extracting rich texture and structural features from plant leaf images, and applying PCA for dimensionality reduction, we ensured that only the most relevant features were fed into the RVFL classifier.Comparative analysis demonstrated that the DenseNet121-RVFL model consistently outperforms in terms of accuracy, AUC and F1-Score, and G-Mean, achieving a top accuracy of 94.45% on the leaf dataset, F1-Score of 0.955, G-Mean value of 0.898 and AUC of 0.961. These results validate the effectiveness of integrating deep feature learning with lightweight, interpretable classifiers, particularly in complex visual recognition tasks such as plant leaf classification.This model currently performed binary classification problems only. Our Future work will explore multi-class and multi-label classification problems in other domains.

## Data Availability

No datasets were generated during the current study. The dataset used in this study is publicly available on Kaggle at: Kaggle Leaf Image Dataset.

## Abbreviations

RVFL: Random vector functional link
CNN: Convolutional neural network
DL: Deep learning
ML: Machine learning
PCA: Principal component analysis
SVM: Support vector machine
TSVM: Twin support vector machine
ELM: Extreme learning machine
KRR: Kernel ridge regression
RBF: Radial basis function
AUC: Area under curve
G-Mean: Geometric mean
KKT: Karush-Kuhn-Tucker conditions
QPP: Quadratic programming problem
